# Supplementary material for: Workplace Factors, Burnout Signs, and Clinical Mental Health Symptoms among Mental Health Workers in Lombardy and Quebec during the First Wave of COVID-19
Source: Int J Environ Res Public Health. 2022 Mar 23;19(7):3806. doi: 10.3390/ijerph19073806 (PMC8997415; doi:10.3390/ijerph19073806)
Supplement: Supplementary file 1 [file ijerph-19-03806-s001.zip › ijerph-1628151-supplementary.pdf]

Table S1. Self-reported narratives: agreement between coders (Cohen's Kappa) and recurrency of themes among the two samples.

	Lombardy		Quebec	
	K	%	K	%
**OS - Organizational support**				
Perceived confusion in procedures	.82	5.9%	0.77	8.9%
Suppression of holidays and work permits	-	0.0%	0.68	4.0%
Perception of lack of "moral" support and authoritarian style	.79	4.3%	0.77	10.9%
Lack of safety support regarding COVID 19	.86	5.4%	0.85	6.4%
Lack of technical support	.91	8.6%	0.72	6.9%
Lack of personnel	.96	6.5%	0.81	11.9%
**JD - Job design**				
Change in role, tasks, and procedure	.85	21.5%	0.64	21.3%
Adjustment to rapid changes	.92	6.5%	0.72	8.9%
Increased workload	.81	6.5%	0.70	16.8%
Increased workload due to covid related tasks	.93	3.8%	0.77	4.5%
Reduced workload and tasks	.62	4.8%	0.59	1.0%
**ED - Emotional distress**				
Fear of catching covid at work	.95	2.8%	0.65	2.2%
Fear of transmitting covid to users	.99	1.4%	0.72	2.2%
Sense of generalized uncertainty towards future	.88	3.3%	0.7	1.6%
Uncertainty of redeployment	-	0.0%	0.47	3.3%
Exhaustion and fatigue	.92	12.4%	0.85	11.9%
**DQ - Perception of decrease in the quality of mental health care**				
General perception of decrease in quality of care	.62	3.3%	0.46	2.7%
Interruption / suspension of interventions / activities	.91	22.0%	0.69	3.5%
Perceived worsening and frailty of users and patients	.98	7.5%	0.74	8.4%
Less implication of family members	.97	0.9%	0.88	1.1%
Worsening of the clinical alliance due to remote contacts	.94	17.5%	0.68	3.3%
Worsening of the clinical alliance due to mask wearing	.99	0.5%	0.75	1.1%
Users' barriers hinder remote contacts	.87	13.4%	0.72	2.0%
**TG - Teamwork and group climate**				
Conflicts in group teamwork	.90	2.4%	0.75	3.3%
Lack of support from colleagues, loneliness	.84	3.8%	0.79	2.7%
Reduced quality of teamwork due to remote contacts	.82	3.3%	0.51	2.5%
**WL - Work-Life balance**				
Difficulties in working with children at home	.99	1.4%	0.69	0.5%
Other work-life balance issues	.86	4.7%	0.55	2.2%
**RS - Restrictions and safety rules**				
Users' frustration towards covid restrictions	.79	1.4%	n-a.	0.0%
Colleagues' difficulty towards covid restrictions	.85	0.9%	0.66	1.1%
Masks and safety procedure discomfort	.92	1.1%	0.89	6.4%
Making users' respecting safety norms	.97	4.3%	0.75	5.9%
